# Reduction of free polysaccharide contamination in the production of a 15-valent pneumococcal conjugate vaccine

**DOI:** 10.1371/journal.pone.0243909

**Published:** 2020-12-10

**Authors:** Yoon Hee Whang, Soo Kyung Kim, Hyeseon Yoon, Seuk Keun Choi, Yeong Ok Baik, Chankyu Lee, Inhwan Lee

**Affiliations:** R&D Center, EuBiologics Co., Ltd., Chuncheon, Republic of Korea; All India Institute of Medical Sciences, Bhopal, INDIA

## Abstract

Glycoconjugate vaccines are vaccines in which a bacterial polysaccharide antigen is conjugated to a carrier protein to enhance immunogenicity by promoting T cell-dependent immune response. However, the free (unreacted) polysaccharides remaining after the conjugation process can inhibit the immunogenicity of a conjugate vaccine. Thus, we aimed to reduce the unbound free polysaccharides in the polysaccharide-protein conjugation process for the development of a new 15-valent pneumococcal conjugate vaccine (PCV15) by varying some factors that may affect the conjugation results such as polysaccharide/protein ratio, polysaccharide size, and concentration of a coupling agent in a conjugation reaction mixture. Concentrations of a coupling agent, carbodiimide (EDAC), and a carrier protein (CRM197) used in PCV15 production, during the conjugation process, had little effect on the content of free polysaccharides. However, the size of the polysaccharide was identified as the critical factor to control the free polysaccharide content, with an inverse relationship observed between the molecular weight of the polysaccharide and the residual free polysaccharide content after conjugation. Based on these results, a new PCV15 with low free polysaccharide contamination was produced and tested for immunogenicity using a rabbit model to show that it induces similar level of immune responses in rabbits compared to a comparator vaccine Prevnar13^®^.

## Introduction

Pneumococci are gram-positive, aerobic diplococci bacteria that are encapsulated, and cause a range of clinical infections from mild diseases such as sinusitis and otitis media to severe diseases such as pneumonia, meningitis, and bacteremia [1]. Overall, pneumococcal diseases are estimated to cause 1.6 million deaths annually worldwide [2]. The pneumococcal capsular polysaccharide is the major virulence factor of these bacteria and is highly diverse with more than 90 different serotypes identified to date [1,3,4]. Disease development and severity are diverse with bacterial serotypes, which vary according to age, geographic area, and time [5,6]. Several pneumococcal vaccines have been developed with various serotypes, which can prevent bacterial infections caused by pneumococcal streptococci, including ear infections, sinusitis, pneumonia, bloodstream infections, and meningitis [1,7].

Currently used pneumococcal vaccines can be divided into two categories, pneumococcal polysaccharide vaccines (PPSV) and pneumococcal conjugate vaccines (PCV) [8]. However, since the polysaccharide antigen is not recognized by T cells, the PPSVs produce antibodies as a B cell response without the help of T cells (T cell-independent immunity) [9–12]. On the other hand, PCVs have been known to induce T cell responses as carrier proteins can promote antigen presentation and activate B cells to promote their differentiation (T cell-dependent immunity) [13,14]. In T cell-dependent immunity, B cells can be differentiated into plasma cells to produce long-lasting antibodies, and subsequently differentiate into memory B cells to provide a boosting effect. Therefore, efforts to develop multivalent conjugate vaccines have been made including Synflorix^TM^, which contains 10 serotypes (1, 4, 5, 6B, 7F, 9V, 14, 18C, 19F, and 23F) and Prevnar^®^, which was first licensed as a 7-valent PCV that covers the seven most frequent serotypes 4, 6B, 9V, 14, 18C, 19F, and 23F in 2000, and later developed as a new 13-valent PCV (Prevnar13^®^), which contains six additional serotypes (1, 3, 5, 6A, 7F, 19A).

A conjugate polysaccharide vaccine consists of a polysaccharide as an antigen, which is conjugated to the carrier protein. Free or unconjugated polysaccharides that are not bound to the carrier proteins also exist in a conjugate vaccine due to incomplete conjugation reaction. Since these free polysaccharides are identical to the polysaccharide only vaccines, they may act as contaminants by interfering the immunological responses induced by polysaccharide-protein conjugates. Thus, it is difficult to achieve increased immunogenicity when the amount of free polysaccharide exceeds that of the conjugated polysaccharide [15–17]. Therefore, the low level of free polysaccharide contents is an important control to guarantee the vaccine immunogenicity.

In this study, we report that the molecular size of polysaccharides affects free polysaccharide contents in our developed 15-valent PCV (PCV15). We also compare the immunogenicity of our PCV15 formulation, with reduced free polysaccharide contents, to a commercial PCV, Prevnar13^®^.

## Materials and methods

### Preparation of polysaccharide and CRM197

All 15 serotypes of pneumococcal polysaccharide (PPS) were produced by fermentation of each of the 15 serotype strains of *Streptococcus pneumoniae* obtained from Culture Collection University of Gothenburg (CCUG). All strains were cultivated in Hemin free media, at 37°C, pH 7.2. Glucose was fed during the cultivation to keep its concentration within 1.5–2% in the cultivating media. After cultivation of *S*. *pneumoniae*, sodium deoxycholate (DOC; Merck, Frankfurt, Germany) was added to a final concentration of 0.1% to the cell broth to lyse the cells, followed by centrifugation at 7000 rpm. The supernatant was then subjected to ultrafiltration/diafiltration (UF/DF) against 10-fold volume of pure water using a 50-kDa or 100-kDa cut-off cassette membrane filter (Hydrosart, Sartocon Ultrafiltration cassettes, Sartorius, Göttingen, Germany), depending on the serotypes. 2% of cetyltrimethylammonium bromide (CTAB; Sigma-Aldrich, Missouri, USA) was added to precipitate the capsular polysaccharide (CPS) and further purification of the CPS were carried out by using Hydroxyapatite chromatography. Final purity of the purified CPS was confirmed by size-exclusion chromatography (SEC) HPLC and the residual CTAB concentrations were controlled below 1ppm by monitoring CTAB concentration using reversed-phase (RP) HPLC [18]. CRM197 carrier protein was purified from fermentation broth of *Corynebacterium diphtheria* obtained from American Type Culture Collection (ATCC) by ion exchange chromatography.

### Polysaccharide fragmentation

A microfluidizer (Microfluidics, Massachusetts, USA) instrument was used to reduce the molecular weight of the pneumococcal polysaccharides, at the concentration of 5mg/mL, by adjusting the number of sample passages and the pressure. Since the physical properties of a polysaccharide vary depending on the serotype, the pressure range of the fragmentation process was 5,000–30,000 psi, and the number of repetitions was set to 1–3 circuits to reduce the molecular weight. The molecular weights of the polysaccharides were calculated using size exclusion chromatography (SEC) equipped with multi angle light scattering (MALS) and refractive index (RI) detectors as described in the literature [19]. Briefly, a TSKgel G5000 PWXL column (Tosoh Bioscience, Tokyo, Japan) was used with 10 mM Phosphate Buffer (pH 7.2) containing 145mM NaCl at a flow rate of 0.5 ml/min for the SEC-MALS. Sample volumes of 100μl were injected at a concentration of 0.25 ~ 0.50 mg/ml. The signal at the 90° angle was analyzed to obtain the weight-average molecular weight using the Astra software (Wyatt Technology, California, USA). Pneumococcal polysaccharides with a reduced molecular weight were then adjusted to 5 mg/mL for the activation reaction.

### Activation of polysaccharide

Pneumococcal polysaccharides with a reduced molecular weight were activated by binding to Adipic acid dihydrazide (ADH; Alfa AESAR, Massachusetts, USA) linker molecules. 1-Cyano-4-dimethylaminopyridinium tetrafluoroborate (CDAP; Alfa AESAR, Massachusetts, USA) in acetonitrile was added to 5 mg/mL of the polysaccharide solution at 1/10 of the volume (50 mg/mL) [20]. After 1 min, 0.21 M of Trimethylamine (TEA; Sigma-Aldrich, Missouri, USA) was added at the same volume of CDAP (50 mg/mL, in acetonitrile). After 3 min, the same volume of 0.5 M ADH with 50 mg/mL CDAP (in acetonitrile) was added to the solution, and the activation reaction was performed for 15 h at 4–10°C on a magnetic stirrer at 200–300 rpm using a magnetic stirrer. After the activation reaction was completed, unbound linker molecules and other reagents were removed by diafiltration with 30-fold volume of purified water using a 10-kDa cut-off membrane filter (UF/DF). Degree of activation was measured by evaluating ADH molecules bound on the polysaccharides using TNBS assay [21]. After filtration, the content of the activated polysaccharide was confirmed by the anthrone assay [22].

### Quantification of polysaccharide and protein

Quantification of polysaccharides was conducted by the anthrone assay, which was modified from the original method described in the literature [22]. Briefly, 2.5mL of 0.2% anthrone solution, prepared by adding 200 mg of anthrone reagent (Sigma-Aldrich, Missouri, USA) into 100mL of 98% sulfuric acid (Daejung, Republic of Korea), was added to 1 mL of the blank, standard, and analyte, and heated for 15 min at 65°C in a dry oven. After the mixtures were cooled at room temperature, they were transferred to a 1-mL cuvette, and the absorbance at 625 nm was measured. In the case of serotype 1, absorbance was measured at a wavelength of 550 nm with a different color from that of the other serotypes. Contents of unconjugated, free polysaccharides were evaluated by measuring the concentration of remaining polysaccharides in the supernatant after precipitating the protein-polysaccharide conjugates with DOC in acidic condition. Protein contents were measured by the Lowry assay as described in the literature [23] using Lowry Assay Kit (Thermo Fisher Scientific, Massachusetts, USA) and unbound, free protein content was monitored by SEC-HPLC.

### Conjugation of activated polysaccharide to CRM197

Activated polysaccharide and CRM197 carrier protein were conjugated in 0.1 M MES buffer (pH 6.0) using EDAC reaction [24]. The conjugation reaction was carried out for 15 h at 4–10°C with gentle mixing using a magnetic stirrer. Unbound CRM197 and other reagents were removed by diafiltration with 10mM Phosphate Buffered Saline (PBS; pH 7.2) using a 100-kDa cut-off membrane filter (UF/DF). After completion of the UF/DF process, the polysaccharide content was confirmed by the anthrone assay as described above.

### Formulation

Final formulation of PCV15 was 10mM PBS with 0.005% polysorbate 80 and 0.25 mg/mL aluminum phosphate. The resulting vaccine composition contained 2.2 μg of each polysaccharide (4.4 μg for 6B), 30–60 μg of the carrier protein CRM197, and 0.125 mg of the aluminum adjuvant for a total volume of 0.5 mL. After formulation, the sample was stored at 4–10°C.

### Animal study

Briefly, 0.5 mL of the formulated 15-valent pneumococcal vaccine was administered to 9-week old male New Zealand white rabbits (n = 3) by intramuscular injection and the same dose of Prevnar13^®^ was injected as a control vaccine. Two boosting injections were performed at two-week intervals after the first injection. Serum samples taken at 6 weeks after immunization were analyzed for antibody and functional antibody levels by enzyme-linked immunosorbent assay (ELISA) and opsonophagocytic assay (OPA). All animal studies were approved by the Institutional Animal Care and Use Committee (IACU) of Daegu-Gyeongbuk Medical Innovation Foundation (approval No.: 18010901–00) according to Animal Protection Law (13023, Jan. 20th, 2015).

### Enzyme-linked immunosorbent assay

The antibody titers in the sera were measured by indirect ELISA as reported previously [25]. In brief, the 15 polysaccharide standards (ATCC) were coated in the wells of the microplate at 100 ng/well, and the titer of serum samples at 6 weeks were measured by diluting the serum from 1/200X to 1/409,600X. The absorbance values measured at 450 nm were plotted in a fourth-order equation, and the dilution factor of the value at which the absorbance was 1 was calculated as an ELISA unit.

### Opsonophagocytic assay

OPA was carried out as described in the literature [26], to measure the functional activity of antibodies in sera. Serially diluted serum samples from each vaccinated rabbit were incubated with pneumococci at room temperature for 30 min and then mixed with HL-60 cells (Korean Cell Line Bank, KCLB No. 10240) and baby rabbit complement (Peel-Freez Biologicals) to allow phagocytosis by HL-60 cells. After a 45-min incubation (37°C, 5% CO_2_), the mixture was spread on 1.5% Todd-Hewitt broth supplemented with yeast extract (THYE) agar plate and incubated for overnight. Colonies of pneumococci were counted using NIST's Integrated Colony Enumerator (NICE) software (National Institute of Standards and Technology, Maryland, USA). Opsonic index was defined as the reciprocal of the serum dilution that kills 50% of bacteria.

### Statistical analysis

For ELISA and OPA, two-tailed t-test was used to confirm the statistical significance of the difference in the serum results between the PCV15 and the Prevnar13^®^ groups; P < 0.05 indicated statistical significance. GraphPad Prism v5.01 (GraphPad, La Jolla, CA, USA) was used to perform the statistical analyses.

## Results

### Free polysaccharide contents in each serotypic conjugates

When the molecular sizes of all 15 serotypes of the polysaccharide were controlled within the similar range (124–254 kDa; Table 1 and S1 Fig) and other factors such as weight ratios of polysaccharide and CRM197 conjugation (1:1) or EDAC concentration (0.05M) in reaction mixtures were fixed, the ratios of free polysaccharides in the conjugates were summarized in Table 1. Serotype 5 conjugate showed the highest free polysaccharide content which was higher than 50%, while others showed 17–42% (Table 1).

**Table 1:** Summary of PS-CRM conjugation reactions and free polysaccharide contents in final conjugates.

Serotype	Ratio of PS: CRM concentration in reaction mixture	EDAC concentration (M)	Molecular weight of PS for conjugation (kDa)	Free polysaccharide content (%)
**1**	1:1	0.05	124	36
**3**	1:1	0.05	142	42
**4**	1:1	0.05	186	26
**5**	1:1	0.05	161	56
**6A**	1:1	0.05	130	18
**6B**	1:1	0.05	173	25
**7F**	1:1	0.05	150	35
**9V**	1:1	0.05	138	20
**11A**	1:1	0.05	201	36
**14**	1:1	0.05	254	34
**18C**	1:1	0.05	241	17
**19A**	1:1	0.05	131	42
**19F**	1:1	0.05	150	38
**22F**	1:1	0.05	166	23
**23F**	1:1	0.05	174	25

### Reduction of the free polysaccharide in the serotype 5 conjugate

Because of the complexity of testing all 15 serotypes, serotype 5, which showed the highest free polysaccharide contamination was first tested for the effects of conjugation variables on the free polysaccharide contents. To identify the factors affecting the content of free polysaccharides, two different sizes of type 5 CPS (high MW: 350kDa, low MW: 180kDa) and different concentrations of EDAC and CRM197 for the conjugation reaction were tested for serotype 5. After each conjugation reaction, the free polysaccharide contents of the final conjugates were compared (Table 2).

**Table 2:** Effects of PS:CRM ratio, EDAC concentration and molecular weights on free polysaccharide contents in serotype 5 conjugates.

Conjugation process Conditions		High MW (350 kDa)				Low MW (180 kDa)			
PS: CRM ratio (w/w)	EDAC concentration (M)	Free polysaccharide Content (%)				Free polysaccharide Content (%)			
		1	2	3	Mean	1	2	3	Mean
1:1	0.05	18	25	21	21	48	55	60	54
1:1.5	0.05	21	15	17	18	52	45	49	49
1:1	0.125	15	16	16	16	64	54	57	58
1:1.5	0.125	15	17	12	15	47	51	52	50

While the conjugates prepared from polysaccharides with a high molecular weight (350 kDa) had 15‒21% of free polysaccharide, conjugates with a low molecular weight (180kDa) contained 49–58% of free polysaccharide contents. Increasing the polysaccharide:CRM ratio from 1:1 to 1:1.5 resulted in approximately 1‒3% of free polysaccharide reduction, and increasing the EDAC concentration by 2.5-fold during the reaction resulted in less than 5% reduction, and it is notable that increasing the molecular size of the polysaccharide by only 2-fold resulted in up to 30% decrease in free polysaccharide contents in the conjugates.

### Reduction of the free polysaccharides in conjugates of 15 serotypes

Based on the serotype 5 conjugate results, we hypothesized that the most crucial factor determining the free polysaccharide content in the PCV is the molecular size of the polysaccharide used for conjugation. To test if this conclusion is valid for all other 14 serotypes, various molecular sizes of each serotype of the polysaccharide were prepared and grouped into three categories, high, intermediate, and low molecular weights. High molecular weight group ranged from 187 to 360 kDa with an average of 255 kDa, intermediate and low size groups ranged from 152 to 305 kDa and 107 to 254 kDa, with average sizes of 211 kDa and 155 kDa, respectively (Table 3).

**Table 3:** Summary of the relationship between molecular weights and free PS contents of all 15 serotype conjugates.

		^1)^HMW			^2)^IMW			^3)^LMW			Free PS (%)		
	
Serotypes	Initial MW	MW	Pressure	No. of Passage	MW	Pressure	No. of Passage	MW	Pressure	No. of Passage	HMW	IMW	LMW
1	340	258	10000	1	201	15000	1	124	30000	1	28	31	36
3	589	210	20000	2	152	30000	2	109	30000	3	28	31	42
4	405	232	5000	1	220	10000	1	186	20000	1	12	16	26
5	289	220	5000	1	181	10000	1	115	30000	1	50	51	56
6A	570	193	5000	1	161	10000	1	108	30000	1	14	15	18
6B	591	240	5000	1	196	10000	1	123	30000	1	19	21	25
7F	675	256	5000	1	220	10000	1	150	30000	1	27	29	35
9V	481	198	10000	1	173	20000	1	138	30000	1	15	19	20
11A	384	287	10000	1	251	20000	1	201	30000	1	24	29	36
14	576	318	10000	1	290	20000	1	254	30000	1	31	34	34
18C	790	360	30000	1	305	30000	2	241	30000	3	13	16	17
19A	372	187	5000	1	161	5000	2	131	5000	3	32	36	42
19F	405	345	5000	1	219	5000	2	167	5000	3	29	36	38
22F	706	317	20000	1	279	30000	1	166	30000	2	9	19	23
23F	721	208	10000	1	156	20000	1	107	30000	1	18	19	25

^1)^High molecular weight group.

^2)^Intermediate molecular weight group.

^3)^Low molecular weight group.

As expected, it was clear that, in all serotypes (1, 3, 4, 5, 6A, 6B, 7F, 9V, 11A, 14, 18C, 19A, 19F, 22F, and 23F), free polysaccharide contents decreased when the sizes of the polysaccharide used for conjugation were increased, i.e., they are in inverse relationship. In the high molecular weight group, the average free polysaccharide content was 23%, whereas in the intermediate and low molecular weight groups, they were 27% and 31%, respectively.

### Immunogenicity *in vivo*

**Fig 1:**
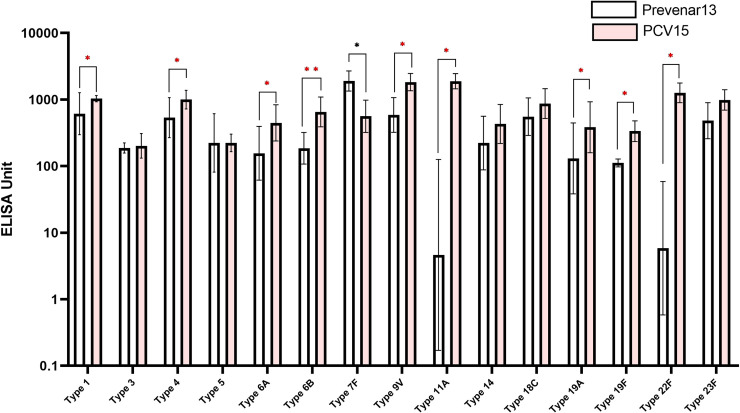
Immune responses against pneumococcal polysaccharides in rabbit measured by ELISA. Prevnar13^®^ was used as a comparator vaccine and PCV15 indicates the new polysaccharide-carrier protein conjugate produced in this study. Serotypes of PCV15 that showed higher ELISA units than Prevnar13^®^ were indicated by red asterisks, and the serotypes with lower were indicated by blue asterisks (*p<0.05, **p<0.01).

PCV produced with 15 polysaccharides of the high molecular weight group, which contained the lowest levels of free polysaccharide contaminants (PCV15), were tested for the immunogenicity in rabbits to compare with a licensed polyvalent pneumococcal conjugate vaccine, Prevnar13^®^. Antibody production, measured by ELISA, from rabbit sera immunized with the PCV15 was higher or equivalent to that obtained with Prevnar13^®^ for 12 (1, 3, 4, 5, 6A, 6B, 9V, 14, 18C, 19A, 19F, 23F) of the 13 serotypes included in Prevnar13^®^ (Fig 1). One serotype (7F) showed relatively low IgG level. The two serotypes (11A and 22F) not included in Prevnar13^®^ showed similar antibody titer to the other 13 serotypes.

**Fig 2:**
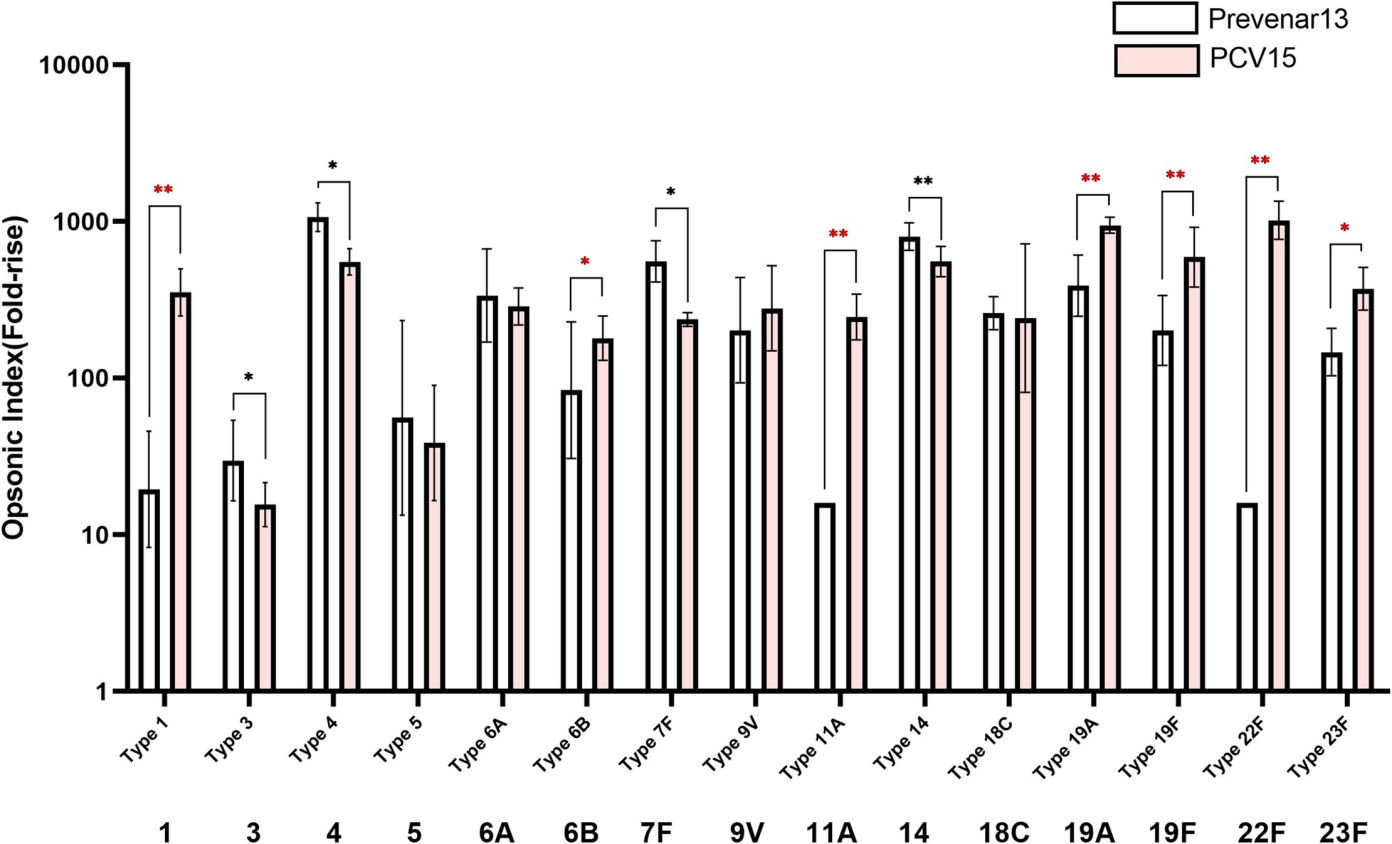
Immune responses against pneumococcal polysaccharides in rabbit measured by OPA. Prevnar13^®^ was used as a comparator vaccine and PCV15 indicates the new polysaccharide-carrier protein conjugate produced in this study. Serotypes of PCV15 that showed higher fold-rises in opsonic index than Prevnar13^®^ were indicated by red asterisks, and the serotypes with lower were indicated by blue asterisks (*p<0.05, **p<0.01).

The OPA assay, which measures the functional antibody in the sera, also demonstrated that PCV15 induces similar levels of immune response compared to Prevnar13^®^ (Fig 2). Seven serotypes (1, 6B, 9V, 18C, 19A, 19F, 23F) in the sera of rabbits immunized with PCV15 demonstrated higher functional antibody levels than those in rabbits receiving Prevnar13^®^, and a low opsonic index was observed for the other six types (3, 4, 5, 6A, 7F, 14). The two serotypes 11A and 22F not included in Prevnar13^®^ showed an opsonic index similar to that of the other 13 serotypes. Overall, the rabbit immunization experiments indicated that the CRM197 conjugate of 15 pneumococcal polysaccharides showed equivalent immunogenicity to that conferred by Prevnar13^®^.

## Discussion

We carried out an optimization of a new pneumococcal conjugate vaccine that contains 15 serotypes (1, 3, 4, 5, 6A, 6B, 7F, 9V, 11A, 14,18C, 19A, 19F, 22F, and 23F) of polysaccharides by adjusting the molecular sizes of the polysaccharides used in the conjugation processes to reduce the free polysaccharide contamination. Among the factors affecting the immunogenicity of PCVs, the content of free polysaccharide, not conjugated to the carrier protein, has a critical impact on the quality of the vaccine since it has a negative effect on the final immunogenicity as the immune responses can be directed to these free polysaccharides rather than to conjugated forms [16,17].

When tested for serotype 5 conjugate, increasing EDAC concentrations in the conjugation reaction did not affect the free polysaccharide contents in the final conjugate product, and it appears that increasing CRM197 by 1.5-fold decreases free polysaccharide by 1‒3%, although negligible. However, we demonstrated an inverse relationship between the molecular weight of the polysaccharide used in the conjugation process and the contents of free polysaccharide, indicating that free polysaccharide could be reduced by controlling the molecular weight of the polysaccharide. We postulated that with a limited amount of CRM197 molecules in a reaction mixture, shorter polysaccharide chains would have more chances to be as unconjugated polysaccharide molecules, than larger polysaccharide chains when equal weights of total polysaccharide are presented. This finding was also supported by the results from other 14 serotypes; however, a polysaccharide with a large size hinders the further process since the viscosity of a polysaccharide solution increases as the size of the polysaccharide increases, leading to a low solubility. Therefore, considering the correlation between the convenience of the pneumococcal polysaccharide conjugation process and the content of free polysaccharide, the optimal molecular weight ranges specific to the polysaccharide per serotype should be compromised with the free polysaccharide contamination to produce a multivalent PCV with adequate immunogenicity.

## Supporting information

S1 Fig SEC-MALS of pneumococcal polysaccharides. Sizes of each serotype of polysaccharides after fragmentation were measured by SEC-MALS. (A) Serotypes 1, 3 4, 5, 6A, (B) 6B, 7F, 9V, 11A, 14, (C) 18C, 19A, 19F, 22F, 23F.
(TIF)
