# Supplementary figures and images for: Reduction of free polysaccharide contamination in the production of a 15-valent pneumococcal conjugate vaccine
Source: PLoS One. 2020 Dec 10;15(12):e0243909. doi: 10.1371/journal.pone.0243909 (PMC7728214; doi:10.1371/journal.pone.0243909)

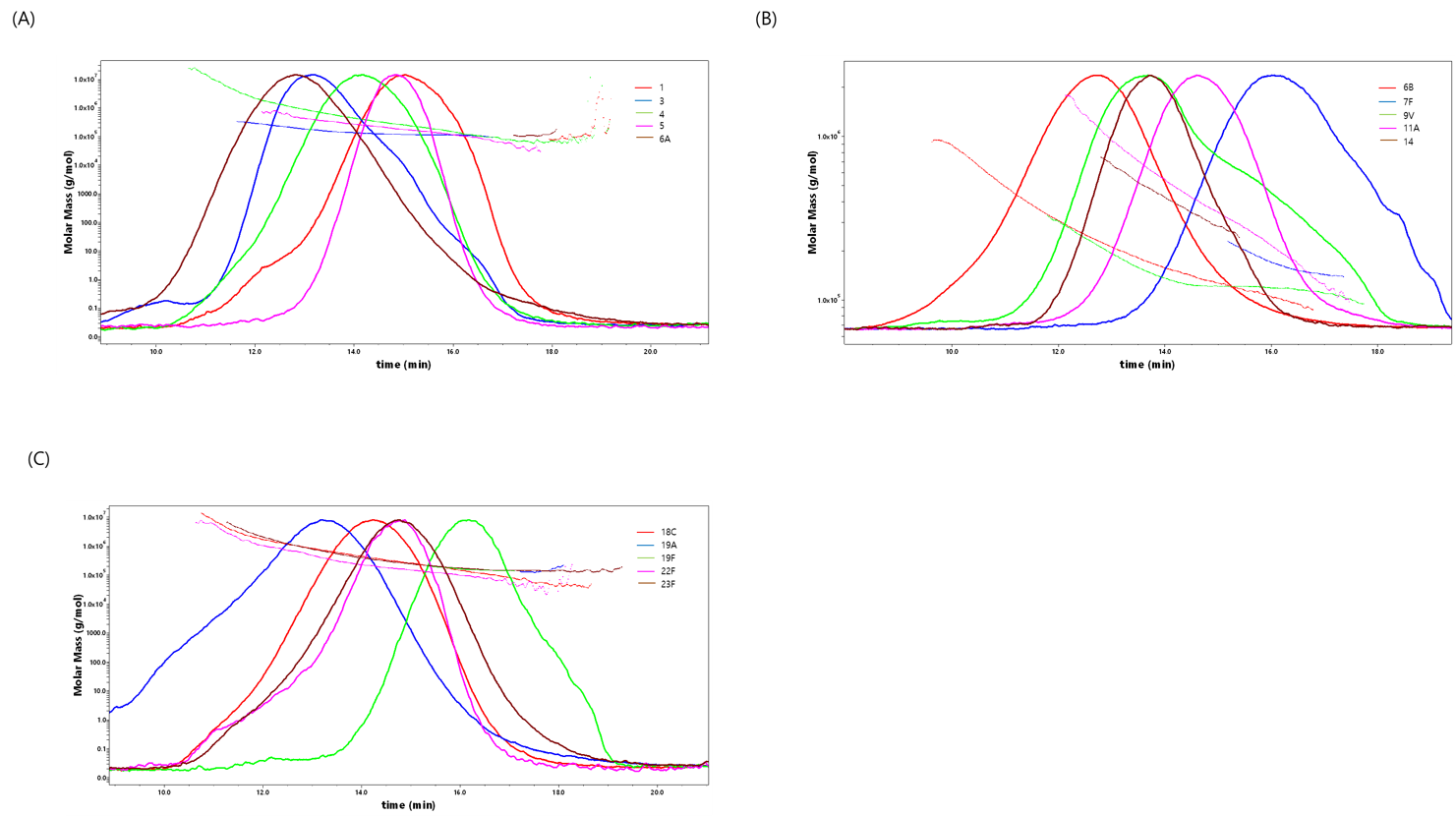

Supplement: S1 Fig — Sizes of each serotype of polysaccharides after fragmentation were measured by SEC-MALS. (A) Serotypes 1, 3 4, 5, 6A, (B) 6B, 7F, 9V, 11A, 14, (C) 18C, 19A, 19F, 22F, 23F. (TIF) [file pone.0243909.s001.tif]
